# CHIP Regulates AKT/FoxO/Bim Signaling in MCF7 and MCF10A Cells

**DOI:** 10.1371/journal.pone.0083312

**Published:** 2013-12-20

**Authors:** Yanrong Lv, Shanshan Song, Kai Zhang, Haidong Gao, Rong Ma

**Affiliations:** 1 Department of Breast Surgery, QiLu Hospital of Shandong University, Jinan, Shandong, China; 2 Department of Molecular Microbiology and Immunology, University of Southern California, Los Angeles, California, United States of America; Sun Yat-sen University Medical School, China

## Abstract

A number of studies have shown that apoptosis resistance can be observed in multiple human tumors; however the detailed mechanism remains unclear. In the present study, we demonstrated that the abnormal overexpression of the C terminus of Hsc70-interacting protein (CHIP) induced apoptosis resistance by regulating the AKT/FoxO/Bim signaling pathway in the breast cancer cell MCF7 and the human non-tumorigenic cell MCF10A. We found that CHIP overexpression in MCF7 and MCF10A cells activated AKT and inhibited the Forkhead box O (FoxO) transcription factors FoxO1, FoxO3, and FoxO4, thereby inhibiting transcription of the target genes *bim* and *pten*. Inhibition of PI3K by a chemical reagent revealed that these events may be critical for CHIP-induced apoptosis resistance. We also determined that inhibition of FoxO3 by CHIP led to the decrease in PTEN and further activated the AKT survival pathway. We corroborated our findings in breast cancer tissues. In general, the CHIP-modulated AKT/FoxO/Bim signaling pathway was shown to induce apoptosis resistance by decreasing the protein level of the tumor suppressor PTEN in both transcriptional and post-translational regulations.

## Introduction

PI3K/AKT signaling has been identified to be deregulated with high frequency in human tumors, especially in breast cancer. Abnormal activated PI3K/AKT signaling can lead to excessive proliferation and resistance to chemotherapy-induced apoptosis. Excessive PI3K/AKT signaling that leads to cell proliferation and defense against apoptosis occurs frequently in breast cancer.

Forkhead box O (FoxO) transcription factors are identified as the essential regulators of multiple cellular activities such as proliferation, defense against oxidative stress, and apoptosis [1]–[2]. These factors are also associated with various human cancers [3]–[4]. Several members of the FoxO family can be regulated by AKT, including FoxO1, FoxO3, and FoxO4 [5]. AKT promotes phosphorylation of these molecules, which leads to their degradation. As FoxO protein is reduced, the target genes involved in apoptosis, such as *puma* and *bim*, become inhibited in transcriptional steps [6]–[8].

The C terminus of Hsc70-interacting protein (CHIP) has previously been identified as a regulator of the human epidermal growth factor receptor 2 (HER2), estrogen receptor-α (ER α), and hypoxia-inducible factor 1α (HIF-1α) [9]–[12]. CHIP has also been known as an essential regulator of apoptosis by modulation of tumor suppressor proteins [13]–[15]. CHIP can protect cells from cellular stress-induced apoptosis [16] and has been identified as a new E3 ligase of phosphatase and tensin homolog deleted on chromosome 10 (PTEN), in addition to Nedd4-1 and WWP2 [17]–[18].

In the current study, the possibility of the occurrence of the CHIP regulating AKT/FoxO/Bim signaling pathway was determined. We demonstrated that CHIP can downregulate the Bim protein level by promoting PTEN ubiquitin-proteasomal degeneration.

## Materials and Methods

All of the human breast cancer samples collected from the Department of Breast Surgery and the Department of Pathology, QiLu Hospital of Shandong University had the approval of the Ethics Committee, QiLu Hospital of Shandong University. The written informed consent from the donors was obtained for use of the samples in research.

### Antibody sources

Antibodies against CHIP, PTEN, normal rabbit immunoglobulin G antibody (normal IgG), and *β*-actin were obtained from Santa Cruz Biotech (USA). Antibodies recognizing FoxO1, p-FoxO1 (S256), FoxO3, p-FoxO3 (S253), FoxO4, p-FoxO4 (S193), AKT, p-AKT (T308), p-AKT (S473), Bim and Ub were purchased from Cell Signal Technology (USA). An antibody against Myc-tag was supplied by Sigma–Aldrich (USA).

### Cell culture and treatment

Human breast cancer MCF7 cells were grown in Dulbecco's modified eagle medium (DMEM) added with 10% fetal bovine serum (Hyclone, USA). Human non-tumorigenic MCF10A cells were maintained in the mammary epithelial growth medium DMEM-F12 (Hyclone, China) containing insulin, hydrocortisone, epidermal growth factor, horse serum, and cholera toxin (Sigma–Aldrich, USA). Cells were incubated at 37°C in a humidified incubator with 5% CO_2_.

The PI3K inhibitor LY294002 and proteasome inhibitor MG132 were purchased from Sigma–Aldrich (USA). Both LY294002 and MG132 were diluted in dimethyl sulfoxide (DMSO). In some experiments, cells were treated with LY294002 or DMSO (Sigma–Aldrich, USA) as control.

### Plasmid transfection

About 1.0×10^5^ cells were seeded into a 12-well plate. Cells were transfected with indicated plasmids by TurboFect in *vitro* transfection reagent (Fermentas, CA) after 24 h, according to the manufacturer's instructions.

### RNA interference and transfection

The following siRNA oligos were purchased from GenePharm (China): CHIP, 5′- AATGAATTCATGGGGATGAAGGGCAAGGAGG-3′; FoxO1, 5′-CCAUGGACAACAACAGAUUGACUCUGA-3′; FoxO3, 5′-GGCUCCUCCUUGUACUCAA-3′; FoxO4, 5′-UGCUUUAACGGGCGGAUAA-3′; PTEN, 5′-GCGCTATGTGTATTATTAT-3′. Cells were cultured in a 12-well plate and transfected with these siRNAs by Lipofectamine 2000 (Invitrogen, USA) in accordance with the protocol.

### Immunoblotting

Cells were washed with cold PBS. The cell lysates were prepared in a TNE buffer (50 mM Tris (pH 7.5), 150 mM NaCl, 1 mM EDTA, and 1% NP-40) and fractioned on 10% to 12% sodium dodecyl sulfate polyacrylamide gel electrophoresis gels. The proteins were transferred to nitrocellulose membranes and then incubated with the respective primary antibodies and corresponding horseradish peroxidase (HRP)-conjugated secondary antibodies (Zhongshan Gold Bridge, China). The bands of the proteins were visualized by chemiluminescence (Thermo, USA).

### Chromatin immunoprecipitation (ChIP)

Chromatin IP Kit was purchased from Cell Signaling Technology (USA). Cells transfected with Myc-CHIP for 48 h were subjected to ChIP with an anti-FoxO3 antibody and a normal IgG antibody. Precipitated DNA was amplified by PCR with *bim*-specific primers or *pten*-specific primers. The PCR products were analyzed by agarose gel electrophoresis and visualized with ethidium bromide under ultraviolet light. Purified DNA was analysed by real-time PCR, using the SYBR Green mix (Bio-Rad, USA) with the MyIQ machine (Bio-Rad, USA). Primers used to amplify the *bim* or *pten* promoter containing the FoxO3 binding site include the following: *bim*: forwards, 5′-AGGCAGAACAGGAGGAGA-3′; reverse, 5′-AACCCGTTTGTAAGAGGC-3′; and *pten*: forwards, 5′-GCATTTCCGAATCAGCTCTCT-3′; reverse, 5′-CCAAGTGACTTATCTCTGGTCTGAG-3′.

### Apoptosis assays

Cells were transfected with Myc-CHIP or negative control vector. After 48 h, the transfected cells were treated with cisplatin (10 µM, 24 h, Sigma–Aldrich, USA). The apoptotic cells were then washed with PBS and stained with fluorescein isothiocyanate-labeled annexin V and propidium iodide (PI) according to the manufacturer's protocol (Promega, USA). Apoptotic cells (Annexin V-positive, PI-negative) were determined by flow cytometry, using Cytomics FC500 (Bekman Coulter, USA).

### RT-PCR

Total RNA was extracted using a Trizol reagent (Invitrogen, USA) according to the manufacturer's instructions. Reverse transcription was performed using ReverTra Ace reverse transcriptase, oligo(dT) primer, and dNTPs (TOYOBO, JP); the resultant cDNA was subjected to PCR. The primer sequences were as follows: PTEN, 5′-ACAGTAGAGGAGCCGTCAAAT-3′ and 5′-TCAGACTTTTGTAATTTGTGT-3′ [2]; GAPDH, 5′-GTATTCCCCCAGGTTTACAT-3′ and 5′-TTCTGTCTTCCACTCACTCC-3′.

### Analysis of CHIP and Bim Expression in Breast Cancer Samples

Proteins in 28 breast cancer tissue samples were obtained from the Department of Breast Surgery, QiLu Hospital of Shandong University. All tissues were extracted by radio immunoprecipitation assay buffer (20 mM Tris (pH 7.5), 150 mM NaCl, 1 mM EDTA, 1 mM EGTA, 1% Triton X-100, 2.5 mM sodium pyrophosphate, 1 mM β-glycerophosphate, 1 mM Na_3_VO_4_, 1 µg/ml leupeptin, and 1 mM PMSF) and then analyzed for CHIP and Bim expression.

### *In vivo* ubiquitylation assay

MCF7 and MCF10A cells were transfected with Myc-CHIP. Twenty-four hours after transfection, cells were treated with 20 µM proteasome inhibitor MG132 for 6 hr. The cells were washed with PBS and lysed in 0.5 ml of HEPES buffer (20 mM HEPES, pH 7.2, 50 mM NaCl, 1 mM NaF, 0.5% Titon-X100) supplemented with 0.1% SDS and protease-inhibitor cocktail (Roche, Germany). The lysates were centrifuged to obtain cytosolic proteins. Briefly, individual samples were incubated with anti-PTEN antibody for 6 h and protein A/G-agarose beads (Santa Cruz) for a further 8 h at 4°C. Then the beads were washed three times with HEPES buffer. The proteins were released from the beads by boiling in 40 µml 2× SDS-PAGE sample buffer for 10 min. The samples were subjected to immunoblotting.

### Immunohistochemical (IHC) staining

Human breast cancer tissues were obtained from the Department of Pathology, QiLu Hospital of Shandong University. The tissues were incubated simultaneously with CHIP (2 µg/ml) and Bim (6 µg/ml) antibodies overnight at 4°C. After washing with PBS, the tissues were visualized using a two-step plus poly-HRP anti-mouse/rabbit IgG detection system (Zhongshan Gold Bridge, China).

### Statistical analysis

Each experiment was repeated at least three times. Statistical significance was evaluated using Student's *t*-test and χ^2^ analysis as required.

## Results

### CHIP-induced apoptosis resistance is associated with AKT/FoxO/Bim signaling

**Figure 1:**
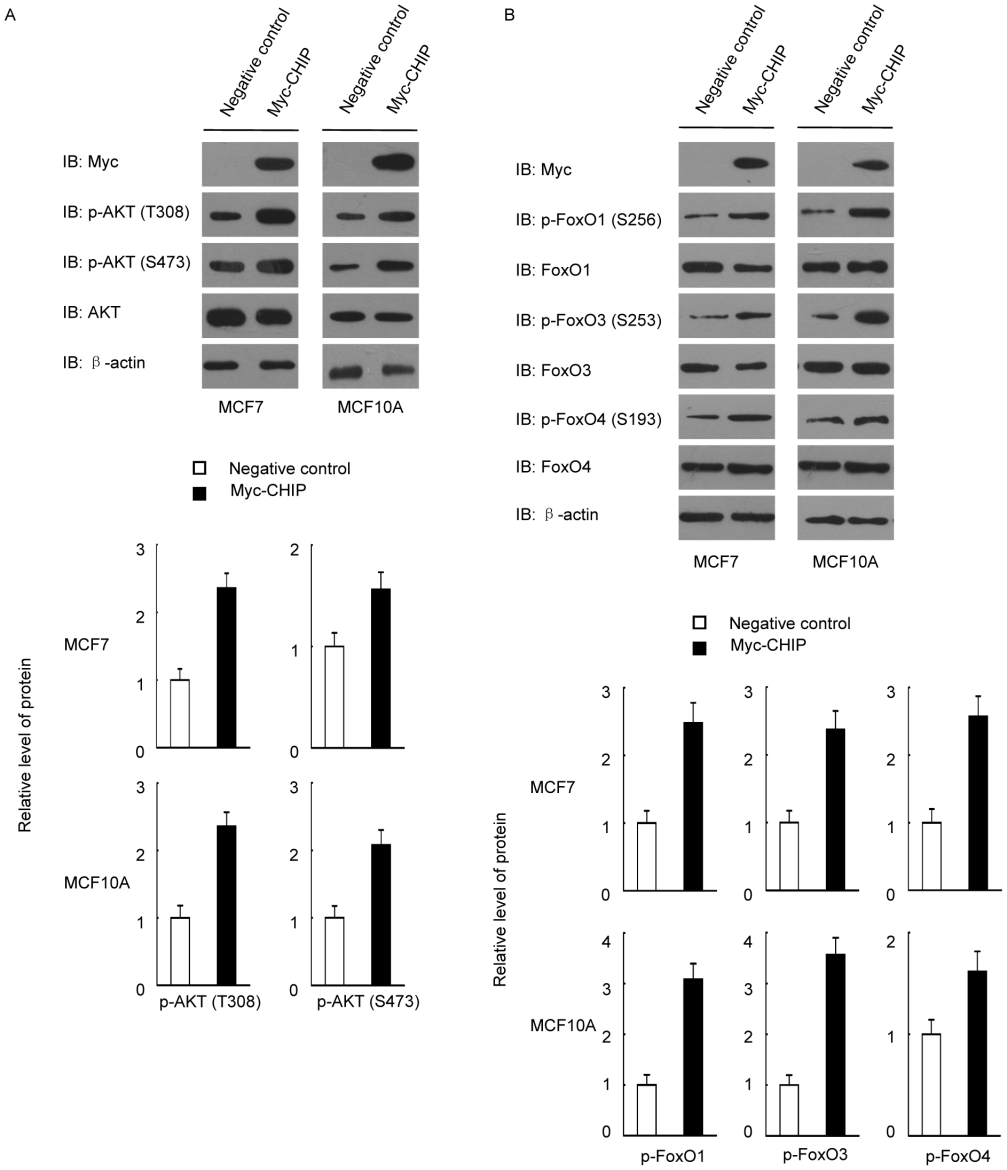
CHIP overexpression activated PI3K/AKT and inhibition of FoxO proteins. (A) CHIP overexpression activated the PI3K/AKT signaling pathway. MCF7 and MCF10A cells were transfected with Myc-CHIP or Myc empty vectors as negative control. The cells were harvested and blotted after 48 h. (B) CHIP overexpression increased the phosphorylation of FoxO family proteins.

Previous studies showed that CHIP protected cells from cell stress-induced apoptosis in multiple cancers. We hypothesized that CHIP significantly affected the AKT/FoxO/Bim axis and then investigated the underlying molecular mechanism. We found that CHIP overexpression promoted PI3K/AKT survival pathway activation in both MCF7 and MCF-10A cells (Figure 1A). This finding suggested that CHIP-activated AKT signaling influences FoxO family members. To prove our hypothesis, we tested phosphorylation of FoxO family proteins in CHIP overexpressed cells. CHIP was shown to significantly promote phosphorylation of these FoxO family proteins (Figure 1B). Thus, FoxO family proteins were inhibited when the AKT pathway was activated by CHIP.

### AKT/FoxO pathway is related to CHIP-induced apoptosis resistance

**Figure 2:**
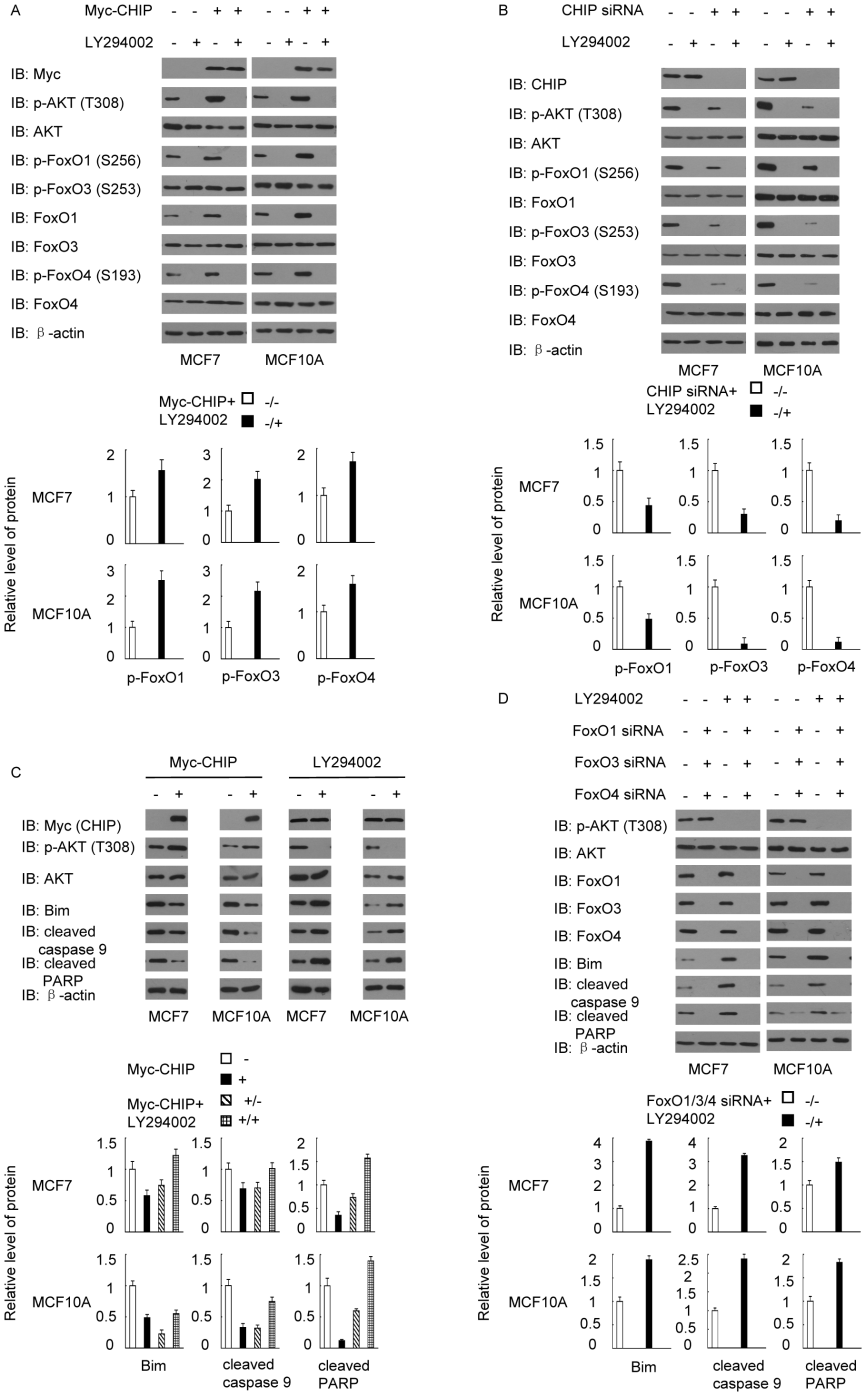
CHIP-induced AKT activation directly inhibited FoxO proteins, an occurrence closely correlated with apoptosis resistance. (A) MCF7 and MCF10A cells were transfected with Myc-CHIP or Myc empty vectors as negative control. AKT inhibition with LY294002 (20 µmol/L, 1h) led to the activation of FoxO proteins. (B) Cells transfected with CHIP siRNA or negative control siRNA were treated with LY294002 (20 µmol/L, 1h) and immunoblotted for p-AKT (T308), p-FoxO1 (S256), p-FoxO3 (S253), and p-FoxO4 (S193). (C) AKT activation induced by CHIP overexpression led to the decrease in Bim, cleaved caspase 9, and cleaved PARP. Treatment with LY294002 (20 µmol/L, 1h) could reverse this effect. (D) Knockdown of FoxO1, FoxO3, and FoxO4 led to the decrease in Bim, cleaved caspase 9, and cleaved PARP. Treatment with LY294002 (20 µmol/L, 1h) could not reverse this effect.

Considering that CHIP enhances PI3K/AKT signaling and inhibits FoxO member proteins, we conducted correspondence experiments to determine the relationship between AKT and the FoxO proteins. When activation of AKT was inhibited by PI3K inhibitor LY294002, CHIP overexpression did not enhance AKT phosphorylation. Minimal AKT phosphorylation led to decreased phosphorylation of FoxO proteins, including FoxO1, FoxO3, and FoxO4 (Figure 2A). Similar results were observed when MCF7 and MCF10A cells were treated with CHIP siRNA (Figure 2B). The results suggested that CHIP-promoted phosphorylation of AKT was related to the phosphorylation of FoxO proteins. We subsequently investigated the interaction of CHIP with AKT/FoxO signaling that induced apoptosis resistance. Western blot was used to test the dose changes of the apoptosis markers between AKT activation and AKT inhibition. The results indicated that when activation of AKT was promoted by CHIP overexpression, the apoptosis markers were reduced, whereas when activation of AKT was inhibited, the apoptosis markers increased significantly (Figure 2C). However, when FoxO1/FoxO3/FoxO4 were silenced by their respective siRNA, AKT inhibition failed to increase apoptosis marker levels (Figure 2D). All results demonstrated that CHIP promoted cell survival by influencing the AKT/FoxO signaling pathway.

### Bim regulated by AKT/FoxO signaling pathway was a key factor participating in apoptosis

**Figure 3:**
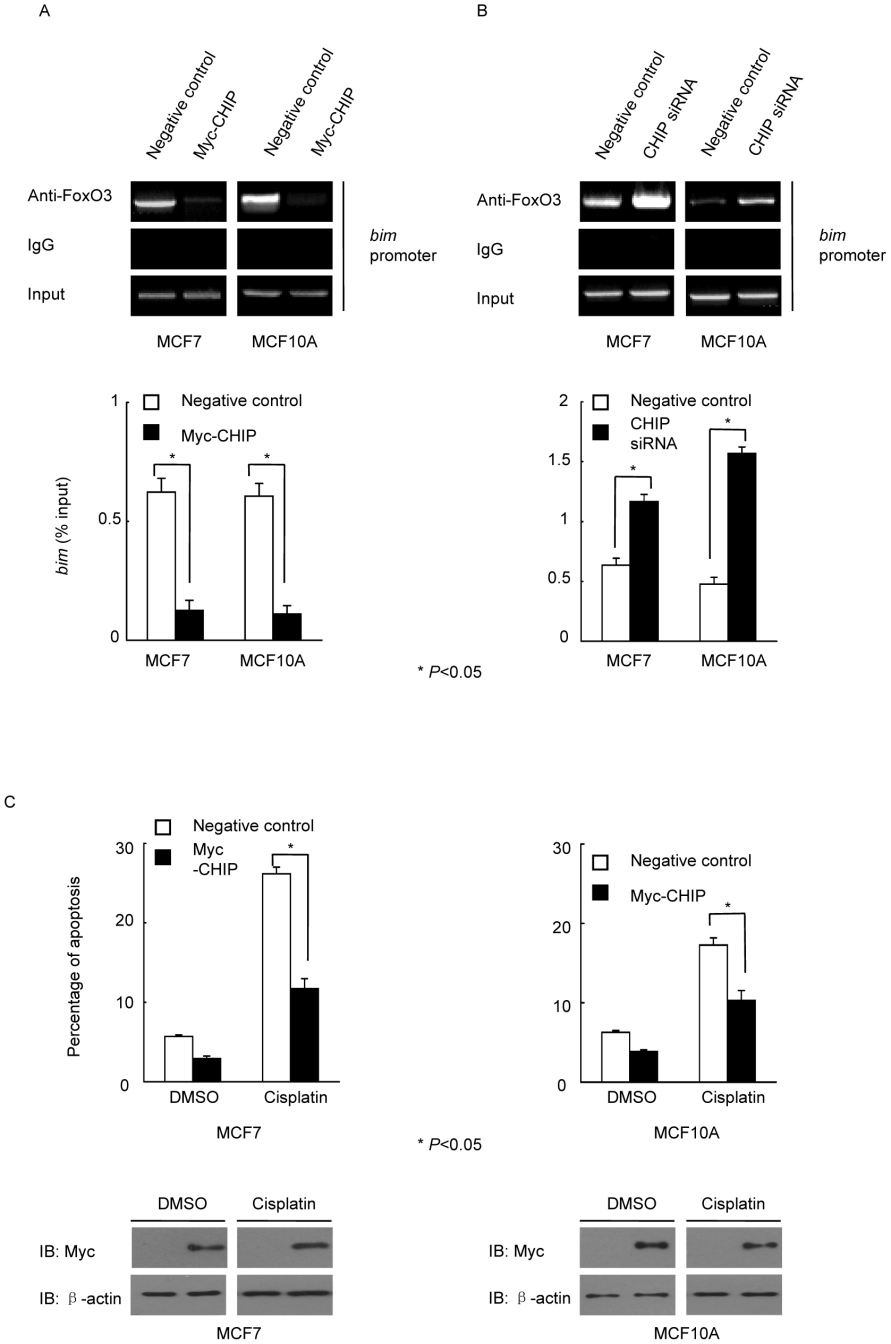
CHIP regulated FoxO3 binding to *bim* and overexpression of CHIP induced apoptosis resistance. (A) CHIP inhibited FoxO3 binding to the *bim* promoter. MCF7 and MCF10A cells were transfected with or without Myc-CHIP, followed by ChIP assay. The relative band intensity of PCR products revealed the binding of FoxO3 to the *bim* promoter. RT-PCR was performed for *bim* promoter (* *P*<0.05). (B) Knockdown of CHIP increased *bim* transcription in both MCF7 and MCF10A cells. Cells were transfected with CHIP siRNA or negative control siRNA, followed by ChIP assay. The relative band intensity of PCR products revealed the binding of FoxO3 to the *bim* promoter. RT-PCR was performed for *bim* promoter (* *P*<0.05). (C) Overexpression of CHIP attenuated apoptosis in cisplatin-treated MCF7 and MCF10A cells. Myc-CHIP was overexpressed in MCF7 and MCF10A cells that were subsequently treated with cisplatin for 24 h. All groups of cells were analyzed by flow cytometry (* *P*<0.05).

FoxO3 can bind to the promoters of *bim*, *puma*, *p21*, and *p27* to promote their transcription [19]. Thus, we tested whether CHIP influenced the binding of FoxO3 to the *bim* promoter and inhibited its transcription. CHIP overexpression in MCF7 and MCF10A cells were shown to reduce the interaction of FoxO3 and the promoter of *bim*; *bim* transcription was inhibited (Figure 3A). Knockdown of CHIP expression with CHIP siRNA in MCF7 and MCF10A cells increased the interaction of FoxO3 and the promoter of *bim*, and *bim* transcription was promoted (Figure 3B). The original data of RT-PCR analysis was shown in Figure S3. We then investigated whether CHIP significantly influenced the apoptosis of MCF7 and MCF10A cells. CHIP was overexpressed in MCF7 and MCF10A cells. Cisplatin-induced apoptosis in MCF7 and MCF10A cells was reduced when CHIP was overexpressed (Figure 3C). The original flow cytometry analysis data was shown in Figure S1 and S2.

### FoxO3 was inhibited by CHIP-downregulated PTEN expression

**Figure 4:**
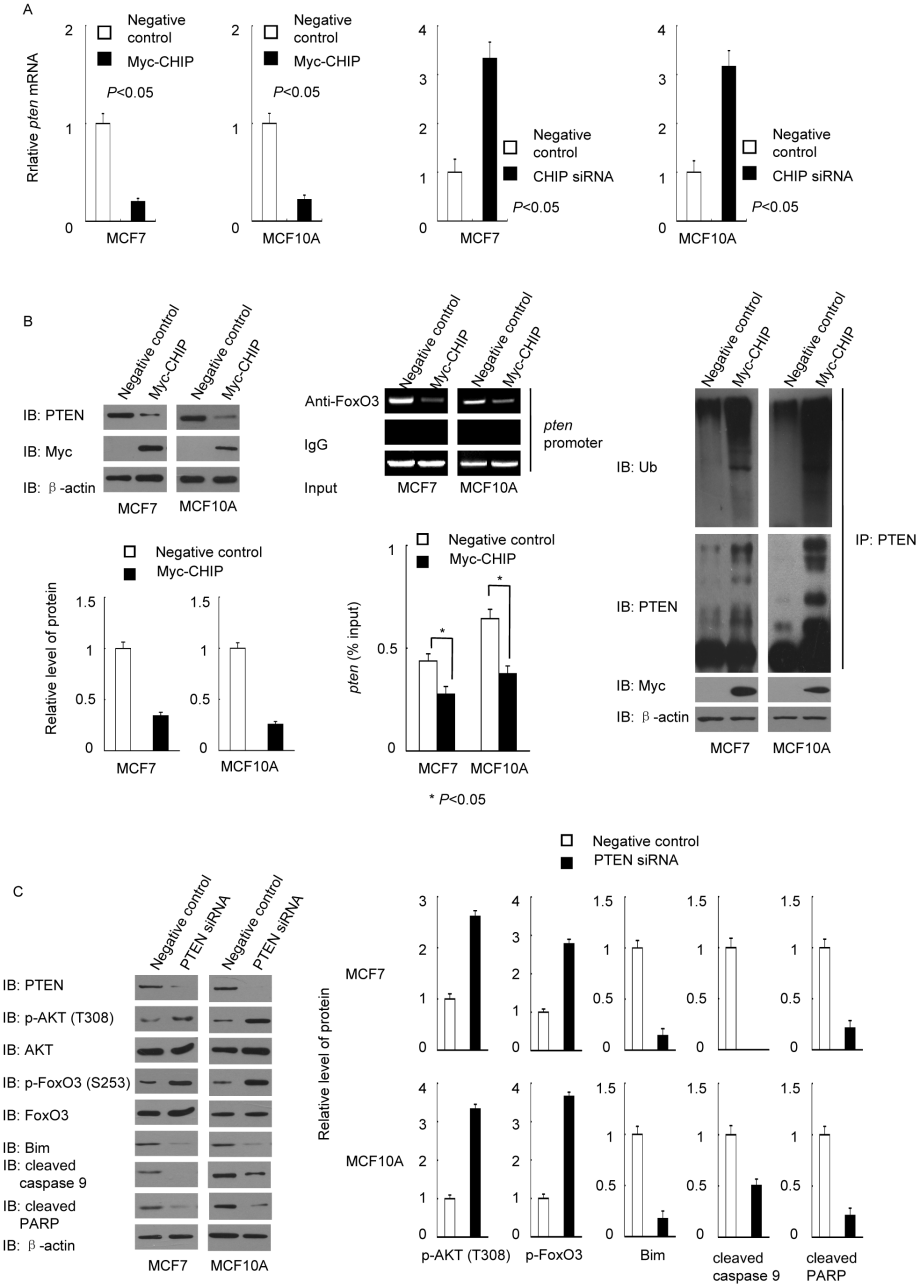
CHIP modulated PTEN, and knockdown of PTEN inhibited the AKT/FoxO3a/Bim signaling pathway. (A) CHIP overexpression reduced the synthesis of PTEN mRNA, and knockdown of CHIP increased PTEN mRNA level. MCF7 and MCF10A cells were transfected with Myc-CHIP, Myc empty vector, CHIP siRNA or negative control siRNA respectively. Total cellular mRNA was then extracted and subjected to reverse transcription PCR. The PTEN mRNA level was determined and calculated from three independent experiments (*P*<0.05). (B) CHIP regulated PTEN protein level in both transcriptional and post-translational steps. MCF7 and MCF10A cells were transfected with or without Myc-CHIP. After 48 h, the cells were treated for western blot, ChIP assay (* *P*<0.05) or *in vivo* ubiquitylation assay respectively. (C) Knockdown of PTEN promoted AKT activation and influenced the AKT/FoxO3/Bim signaling pathway. MCF7 and MCF10A cells were transfected with PTEN siRNA or negative control siRNA. The altered expression patterns of p-AKT (T308), AKT, p-FoxO3, FoxO3, Bim, cleaved caspase 9, and cleaved PARP were determined by western blot.

FoxO3 has been reported to be capable of binding to the promoter of *pten* to promote its transcription [20]. Thus, we hypothesized that a decrease in FoxO3 induced by CHIP overexpression can inhibit the *pten* transcription. To verify this hypothesis, we tested PTEN mRNA level when CHIP was overexpressed or knocked down. PTEN mRNAs decreased sharply when the activity of FoxO3 was inhibited by overexpressed CHIP, while PTEN mRNAs increased significantly when CHIP was deficient (Figure 4A). Previous research also showed that CHIP was an E3 ligase of PTEN [21]. We tested whether overexpression of CHIP could both influence the binding of FoxO3 and the promoter of *pten* and the ubiquitylation of PTEN. The results showed that overexpression of CHIP not only decreased FoxO3 binding to *pten* promoter to inhibit *pten* transcription, but also promoted the ubiquitylation of PTEN to promote PTEN degeneration through ubiquitin-proteasomal pathway (Figure 4B). Thus, CHIP can regulate PTEN in both transcriptional and post-translational stages. To explore whether PTEN downregulation could affect AKT/FoxO signaling, we knocked down PTEN in verify MCF7 and MCF10A cells and tested the changes in proteins in the AKT/FoxO signaling pathway (Figure 4C). The changes in this pathway demonstrated that a decrease in PTEN could promote AKT activation to inhibit the FoxO signaling and lead to apoptosis resistance. CHIP, AKT/FoxO and PTEN may verify a feedback loop to regulate cell apoptosis.

### CHIP regulated the AKT/FoxO/Bim pathway *in vivo*

**Figure 5:**
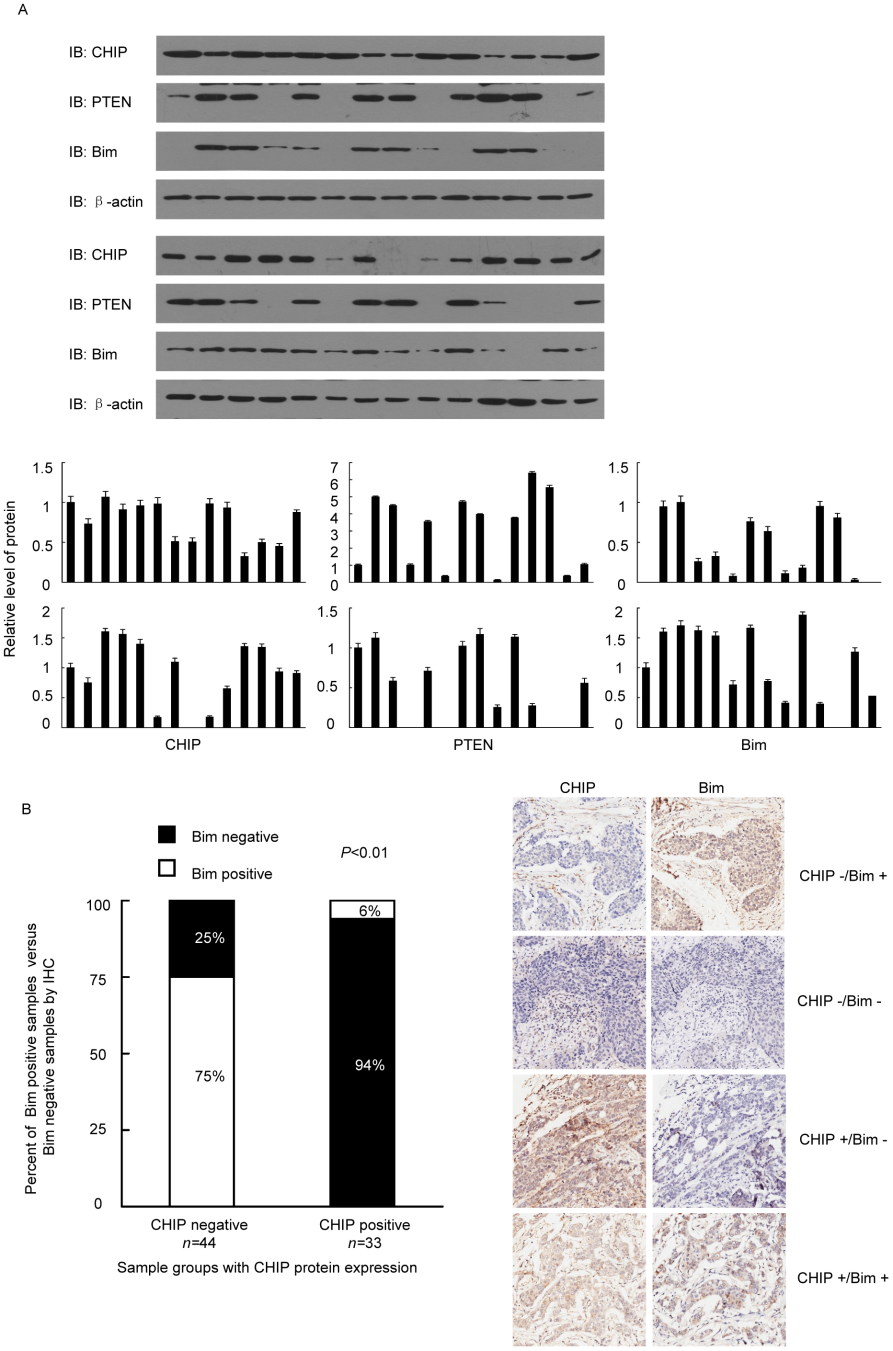
CHIP regulated the AKT/FoxO/Bim pathway in human breast cancer tissues. (A) The relationship of CHIP, PTEN, and Bim protein expression in 28 breast cancer tissue samples. All tissues were analyzed for CHIP PTEN and Bim expression by western blot. (B) IHC of breast cancer tissues for CHIP and Bim analysis and quantification (*n* = 77; *P*<0.01, χ^2^ test).

To confirm our hypothesis, we collected breast cancer tissues from 28 breast cancer patients. The protein levels of CHIP, PTEN, and Bim were tested. The level of Bim positively correlated to that of PTEN but exhibited significant negatively correlation to that of CHIP (Figure 5A). These findings suggested that CHIP is a key factor in the apoptosis resistance of breast cancer cells. We also tested CHIP and Bim expression in 77 breast cancer samples and found that CHIP and Bim were negatively correlated in most samples (Figure 5B).

## Discussion

Our study demonstrated that CHIP-induced apoptosis resistance is closely associated with the AKT/FoxO3/Bim signaling pathway in both normal breast epithelial cell MCF10A and breast cancer cell MCF7 cells. All findings suggested that CHIP overexpression enhances AKT/PI3K signaling and leads to the inactivation of FoxO family members. Further experiments proved that genetically inhibiting CHIP can lead to FoxO3 and Bim activation. Our study also revealed that CHIP-induced inactivation of FoxO3 can inhibit the transcription of *bim* and *pten* by decreasing the binding on the *FoxO3* promoter. Reduced Bim protein level inhibited caspase 9 and PARP activation and induced cell apoptosis resistance.

FoxO family members are master regulators that prevent excessive cell activity by controlling different gene expression profiles in response to stimuli [22]. Another study revealed the essential function of FoxO3 in inhibiting differentiation of AML cells [23], which varies from its classic function as a tumor suppressor. Cells could be regulated by multiple upstream regulators such as AKT and JNK. In the present study, we focused on the influence of CHIP-induced AKT phosphorylation on FoxO3 and its downstream molecules because excessive CHIP expression and abnormal AKT activation often occur in multiple human tumors, especially in breast cancers. Thus, determining the main molecular mechanism of CHIP-enhanced AKT activation is important for malignant tumor therapy. We initially found that CHIP promoted AKT activation. We demonstrated that AKT activation inactivated FoxO3, an essential occurrence in apoptosis resistance when cells were treated with chemotherapeutic drugs. The AKT/FoxO3 signaling pathway can be regulated by multiple chemotherapeutic drugs such as 18β-glycyrrhetinic acid and isoflavone [24]–[25]. FoxO3 can be phosphorylated by activated AKT at the Thr32, Ser256, and Ser319 sites. Phosphorylation of these sites promotes FoxO3 binding to 14-3-3 proteins, which can retain FoxO3 in the cytoplasm and inhibit its function in nucleus. Excessive AKT activation by CHIP was also shown to be related to the increased phosphorylation level of FoxO3, which can inhibit FoxO3 by reducing specify dose in the nucleus.

Bim is an important pro-apoptotic molecule and induces cell apoptosis by interacting with anti-apoptotic proteins. The interactions can release Bax and Bak to initiate apoptosis [26]–[27]. The present study indicated that FoxO3 could bind to the *bim* promoter to enhance its transcription.

**Figure 6:**
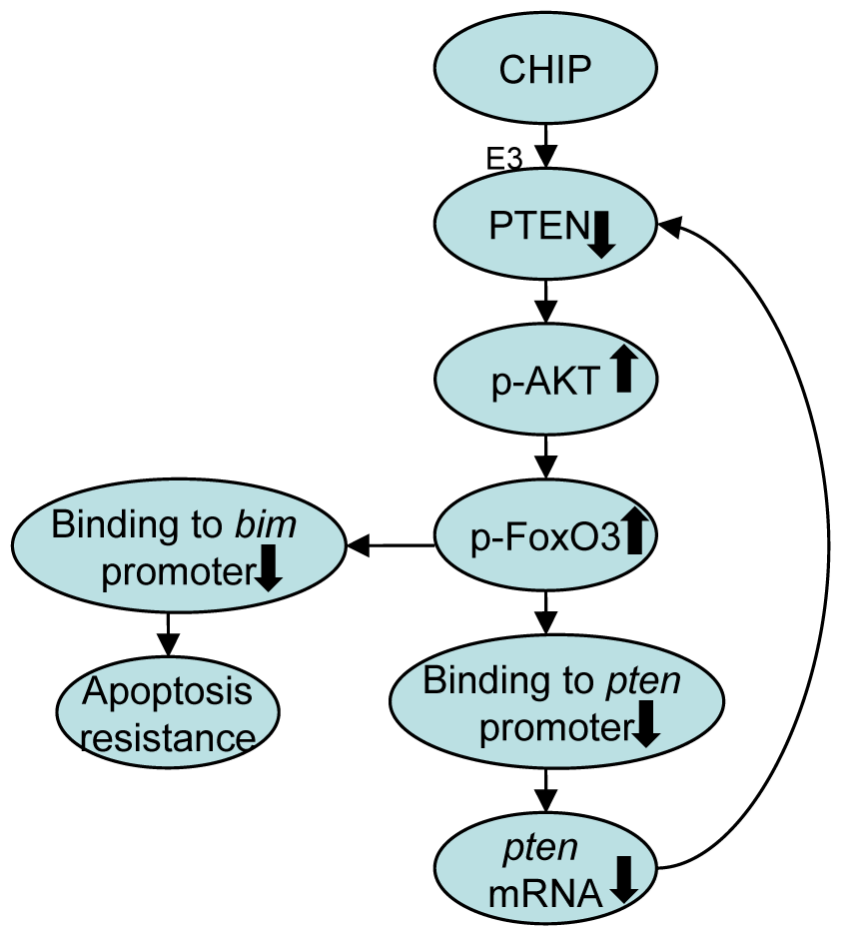
A model described how CHIP regulated PTEN protein level. Overexpression of CHIP promoted degeneration of PTEN, which can increase the activation of PI3K pathway to promote the phosphorylation of FoxO3. FoxO3 binding to bim and pten promoters was inhibited and Bim and PTEN protein levels were decreased. Cells demonstrated apoptosis resistance, and the phosphorylation of AKT increased significantly. It formed a loop to reduce the PTEN protein level constantly.

PTEN is one of the most important tumor suppressors often occurring at a lower protein level in breast cancer tissues than in normal breast tissues. PTEN has been known as a key negative regulator of the AKT/PI3K pathway. Recent research proposed that CHIP is one of the E3 ligases of PTEN and decreases PTEN dose [21]. This finding is supported by our study. However, we also found that CHIP indirectly reduced the mRNA level of PTEN. The mechanism involved the inhibition of FoxO3 by CHIP, resulting in inhibited *pten* transcription. We concluded that CHIP-induced PTEN deficiency was associated with both the ubiquitin–proteasome pathway and the AKT/FoxO3/Bim pathway (Figure 6). We supposed that a positive feed-back loop worked as below: when CHIP worked as an E3 of PTEN, the protein level of PTEN got lower, which can increase the activation of PI3K pathway to promote the phosphorylation of FoxO3 to inhibit its activity. Thus, FoxO3 binding to bim and pten promoters was inhibited and Bim and PTEN protein levels were decreased. When Bim was deficient, cells demonstrated apoptosis resistance. But when PTEN was deficient, the phosphorylation of AKT increased significantly. It formed a loop to reduce the PTEN protein level constantly. Nonetheless, the existence of a real feedback loop between PTEN and the AKT/FoxO3/Bim signaling pathway remains undetermined.

All results of the present study demonstrated that CHIP could induce apoptosis resistance in MCF7 and MCF10A cells by activating PI3K/AKT survival factors and inhibiting FoxO proteins, thereby decreasing Bim and PTEN. These findings elucidated the detailed molecular mechanisms of CHIP-induced apoptosis resistance in human breast cancer cell MCF7 and human non-tumorigenic cell MCF10A and provided a theoretical basis for clinical therapy of breast cancer.

## Supporting Information

Figure S1 The original flow cytometry analysis data of cisplatin induced apoptosis in CHIP overexpressed MCF7 cells.
(TIF)

Figure S2 The original flow cytometry analysis data of cisplatin induced apoptosis in CHIP overexpressed MCF10A cells.
(TIF)

Figure S3 The RT-PCR analysis original data of mRNA levels in MCF7 and MCF10A cells with overexpressed or deficient CHIP.
(TIF)
