# Supplementary figures and images for: CHIP Regulates AKT/FoxO/Bim Signaling in MCF7 and MCF10A Cells
Source: PLoS One. 2013 Dec 20;8(12):e83312. doi: 10.1371/journal.pone.0083312 (PMC3869759; doi:10.1371/journal.pone.0083312)

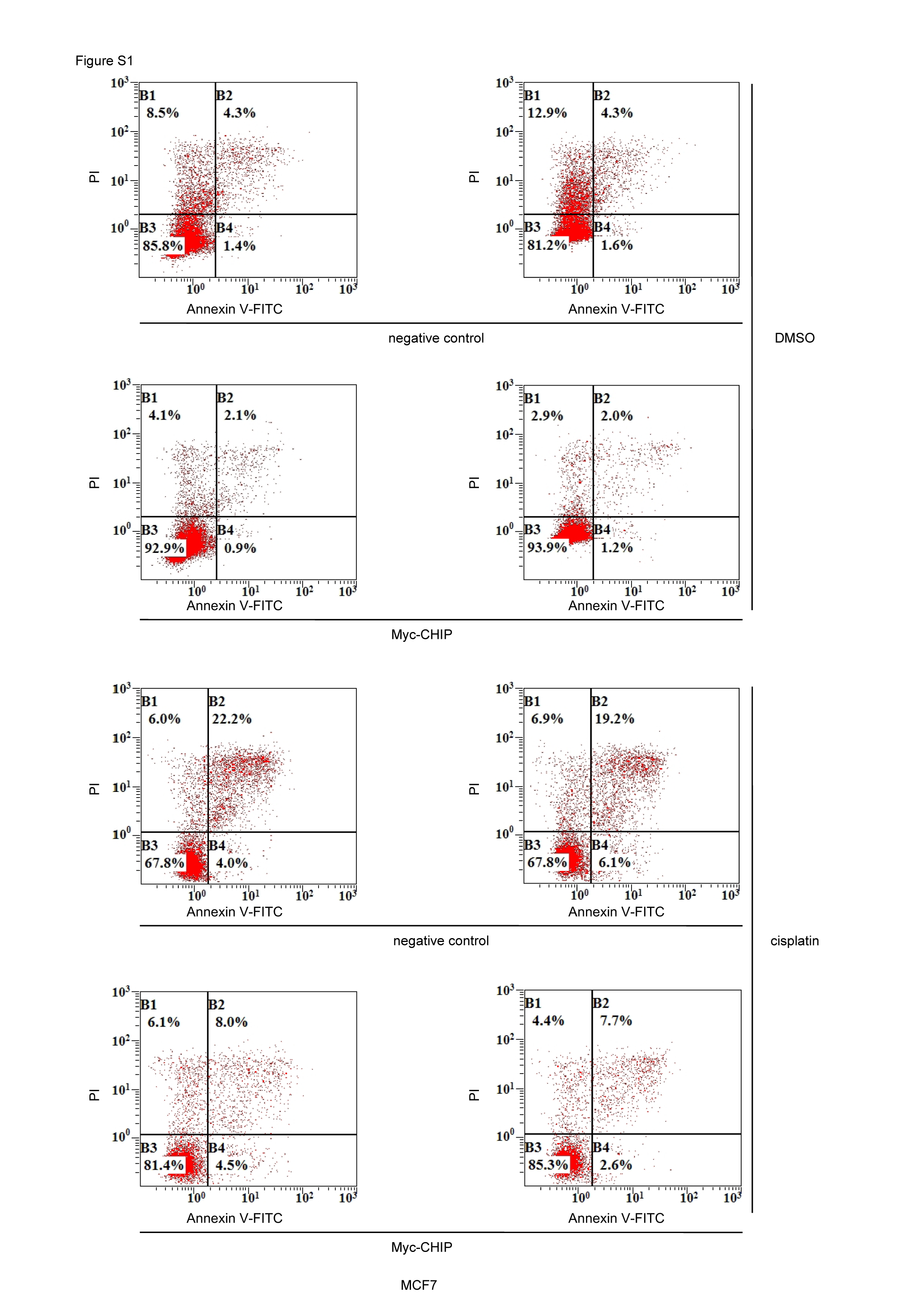

Supplement: Figure S1 — The original flow cytometry analysis data of cisplatin induced apoptosis in CHIP overexpressed MCF7 cells. (TIF) [file pone.0083312.s001.tif]

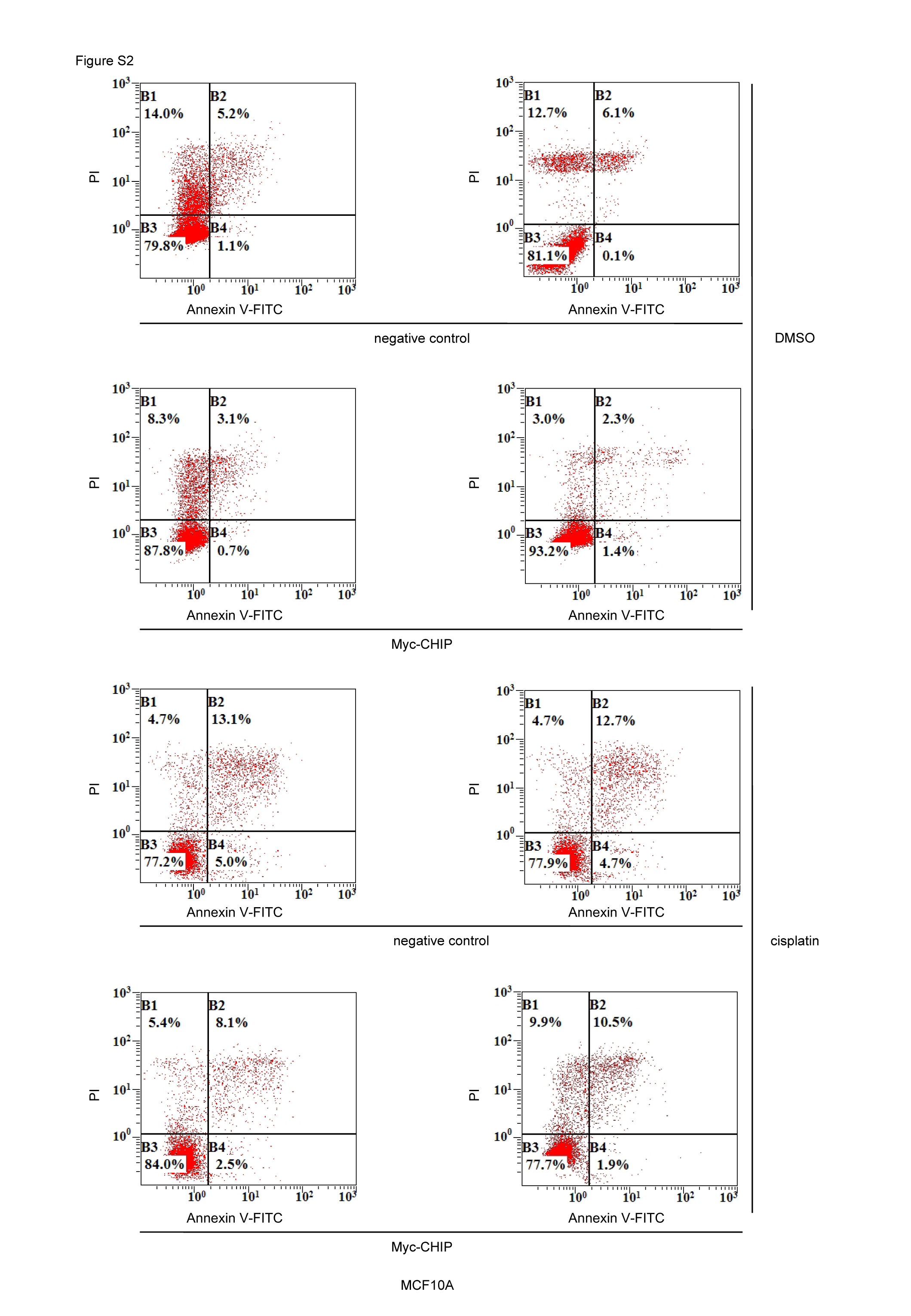

Supplement: Figure S2 — The original flow cytometry analysis data of cisplatin induced apoptosis in CHIP overexpressed MCF10A cells. (TIF) [file pone.0083312.s002.tif]

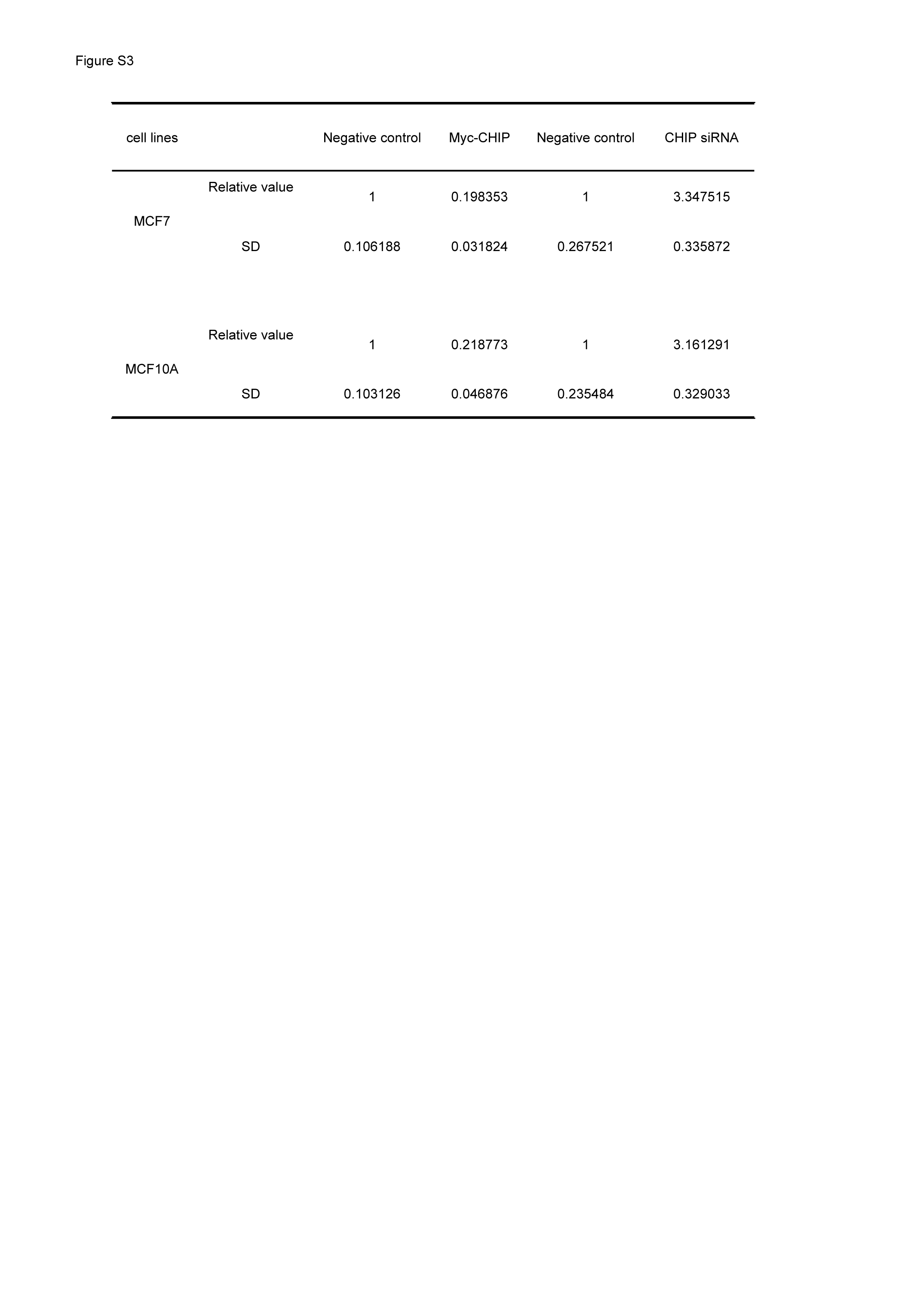

Supplement: Figure S3 — The RT-PCR analysis original data of mRNA levels in MCF7 and MCF10A cells with overexpressed or deficient CHIP. (TIF) [file pone.0083312.s003.tif]
